# Draft Genome Sequence of *Buttiauxella* sp. Strain A111, Which Converts 2-Azahypoxanthine to 2-Aza-8-Oxohypoxanthine

**DOI:** 10.1128/MRA.00664-19

**Published:** 2019-07-18

**Authors:** Jae-Hoon Choi, Ryota Moriuchi, Apirati Sukprasitchai, Shinji Tokuyama, Hirokazu Kawagishi, Hideo Dohra

**Affiliations:** a Research Institute of Green Science and Technology, Shizuoka University, Shizuoka, Japan; b College of Agriculture, Academic Institute, Shizuoka University, Shizuoka, Japan; c Graduate School of Integrated Science and Technology, Shizuoka University, Shizuoka, Japan; d Graduate School of Science and Technology, Shizuoka University, Shizuoka, Japan; University of Maryland School of Medicine

## Abstract

We report here the draft genome sequence of *Buttiauxella* sp. strain A111, isolated on the basis of bioconversion activity of the plant growth-regulating compound 2-azahypoxanthine to 2-aza-8-oxohypoxanthine. The genome contains 4,388 protein-coding sequences, including several genes possibly involved in the metabolism of the plant growth-regulating compound.

## ANNOUNCEMENT

The efficient production method of 2-aza-8-oxohypoxanthine (AOH), a strong plant growth stimulator (1), is limited to bioconversion from 2-azahypoxanthine (AHX) by incubation with resting cells of Burkholderia contaminans CH-1 (2). We isolated a *Buttiauxella* sp. bacterium (designated strain A111) from forest soil in Hokkaido, Japan, by screening based on the bioconversion activity from AHX to AOH. The genus *Buttiauxella* is a member of the family Enterobacteriaceae, which contains an endophytic bacterium, *Buttiauxella* sp. strain SaSR13, that improves the growth of a plant of the genus *Crassulaceae* (3).

**FIG 1:**
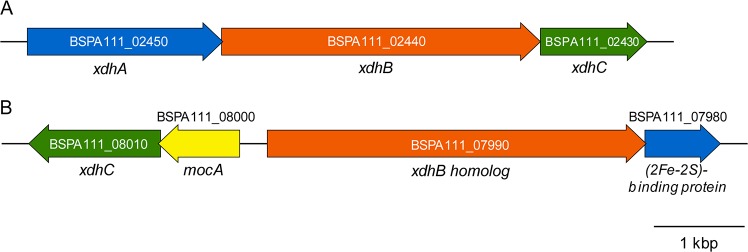
Gene clusters coding for xanthine dehydrogenases of *Buttiauxella* sp. strain A111. (A) The xanthine dehydrogenase *xdhABC* gene cluster. (B) The gene cluster encoding putative xanthine dehydrogenase homologs. Genes *xdhA*, *xdhB*, *xdhC*, and *mocA* encode xanthine dehydrogenase small subunit, xanthine dehydrogenase large subunit, xanthine dehydrogenase accessory factor, and molybdenum cofactor cytidylyltransferase, respectively. Note that the gene coding for the 2Fe-2S-binding protein is shorter than *xdhA* because its product lacks flavin adenine dinucleotide (FAD)-binding and carbon monoxide (CO) dehydrogenase flavoprotein C-terminal domains.

Genomic DNA of *Buttiauxella* sp. strain A111 was extracted using a DNeasy blood and tissue kit and fragmented using a Covaris acoustic solubilizer. A library constructed using a TruSeq DNA PCR-free library preparation kit was sequenced using the Illumina MiSeq platform to generate 301-bp paired-end reads. The raw reads were cleaned up as described previously (4) using Trimmomatic ver. 0.36 (5). The resultant 1,592,778 high-quality read pairs totaling 918 Mb and representing 187.8-fold coverage of the genome were assembled using SPAdes ver. 3.13.0 (6), with the same parameters as reported previously (4). The assembly generated 37 contigs (>200 bp), with a longest sequence of 1,350,757 bp and an *N*_50_ value of 643,281 bp. The draft genome sequence of *Buttiauxella* sp. strain A111 consisted of 4,889,549 bp, with a G+C content of 50.3%. The genome was annotated using the DFAST-core ver. 1.2.0 (7). The genome contains 4,388 protein-coding sequences, 4 rRNA genes, and 79 tRNA genes. Although the 16S rRNA gene of strain A111 showed a high similarity (99.8%) to that of Buttiauxella gaviniae (GenBank accession number NR_025330), average nucleotide identity (ANI) analysis (8, 9) showed the highest ANI value (86.42%) with the Buttiauxella ferragutiae ATCC 51602 genome (GenBank accession number LXEQ00000000). This ANI value was significantly lower than the species threshold of 95% (8), suggesting that *Buttiauxella* sp. strain A111 was a novel species belonging to the genus *Buttiauxella*. The proteomes of *Buttiauxella* sp. strain A111 and *B. contaminans* CH-1 (GenBank accession numbers AP018357 to AP018360) (4) were annotated using KofamKOALA (10) to compare xanthine dehydrogenase (XDH) homologs as candidate enzymes involved in the conversion of AHX to AOH. The *Buttiauxella* sp. strain A111 genome contains two gene clusters coding for XDHs, XdhABC (BSPA111_02450 to 02430) (Fig. 1A), and the xanthine dehydrogenase family protein molybdopterin-binding subunit (BSPA111_07990) and (2Fe-2S)-binding protein (BSPA111_07980) (Fig. 1B). Unlike *B. contaminans* CH-1, molybdenum cofactor cytidylyltransferase MocA (BSPA111_08000) and xanthine dehydrogenase accessory protein XdhC (BSPA111_08010) were encoded adjacent to the upstream area of the latter gene cluster in the *Buttiauxella* sp. strain A111 genome (Fig. 1B) and have been reported to be involved in the cytidylation of molybdenum cofactor (11) and insertion of the cofactor into XDH (12), respectively. It remains to be elucidated whether the high conversion activity in *Buttiauxella* sp. strain A111 is due to the cytidylation of the cofactor. The genome information of *Buttiauxella* sp. strain A111 would be expected to provide important clues as to the biosynthetic pathways and functions of the plant growth-regulating compounds.

### Data availability.

The raw reads of *Buttiauxella* sp. strain A111 have been deposited in the DDBJ Sequence Read Archive under the accession number DRA008321. The draft genome sequence has been deposited in DDBJ/ENA/GenBank under the accession number BJFN00000000.
